# Supplementary material for: Getting More Out of Biomedical Documents with GATE's Full Lifecycle Open Source Text Analytics
Source: PLoS Comput Biol. 2013 Feb 7;9(2):e1002854. doi: 10.1371/journal.pcbi.1002854 (PMC3567135; doi:10.1371/journal.pcbi.1002854)
Supplement: Dataset S1 — GATE software. Dataset S1 bundled with this paper contains a distribution of GATE (or see http://gate.ac.uk/download/). (TGZ) [file pcbi.1002854.s001.tgz › plos-gate/plugins/Tagger_MetaMap/doc/info/olteanu/interfaces/StringFilter.html]

StringFilter


---


|  |  |  |
| --- | --- | --- |
| **Overview** \| **Package** \| **Class** \| **Use** \| **Tree** \| **Deprecated** \| **Index** \| **Help** | |  |
| PREV CLASS   NEXT CLASS | **FRAMES**    **NO FRAMES**     **All Classes** |  |
| SUMMARY: NESTED \| FIELD \| CONSTR \| METHOD | DETAIL: FIELD \| CONSTR \| METHOD |  |


---


## info.olteanu.interfaces Interface StringFilter

**All Known Implementing Classes:**: TextNormalizer.Java5Normalizer, TextNormalizer.Java6Normalizer

---

``` public interface StringFilter ```

---

| **Field Summary** | |
| --- | --- |
| `static StringFilter` | `INVALID` |
| `static StringFilter` | `VOID` |


| **Method Summary** | |
| --- | --- |
| `java.lang.String` | `filter(java.lang.String input)` |

**Field Detail**

### VOID

```
static final StringFilter VOID
```

---


### INVALID

```
static final StringFilter INVALID
```


**Method Detail**

### filter

```
java.lang.String filter(java.lang.String input)
```


---


|  |  |  |
| --- | --- | --- |
| **Overview** \| **Package** \| **Class** \| **Use** \| **Tree** \| **Deprecated** \| **Index** \| **Help** | |  |
| PREV CLASS   NEXT CLASS | **FRAMES**    **NO FRAMES**     **All Classes** |  |
| SUMMARY: NESTED \| FIELD \| CONSTR \| METHOD | DETAIL: FIELD \| CONSTR \| METHOD |  |


---
